# Alcohol and the Hospitals

**Published:** 1901-07-06

**Authors:** 


### THE INSTITUTIONAL WORKSHOP.

### ALCOHOL AND THE HOSPITALS.

By a Correspondent.

In the *Medical Temperance Review* for June the following passage occurs in an editorial entitled "The Cost of Drink in London Hospitals," professing to represent the views of some 500 members of the medical profession. After commenting on the decreased expenditure on alcohol shown in London hospitals during the last thirty years, the editor, Dr. J. J. Ridge, proceeds:—

"But the serious part of the matter is the great and extraordinary difference in the amount consumed at the different hospitals. . . . Let us put it in tabular form as far as the figures allow. Thus the expenditure (*whether per bed or per patient does not appear, but is irrelevant for the present purpose*) [the italics are ours] was as follows:—

Table A.

| | £ | s. | d. |
|---|---|---|---|
| University College Hospital | 0 | 0 | 8¾ |
| London Hospital | 0 | 1 | 7[illegible] |
| Charing Cross Hospital | 0 | 2 | 2¾ |
| St. George's Hospital | 0 | 3 | 0¼ |
| King's College Hospital | 0 | 13 | 6¾ |
| Guy's Hospital | 0 | 13 | 8¾ |
| Westminster Hospital | 0 | 13 | 11½ |
| St. Bartholomew's Hospital | 1 | 2 | 0 |
| Middlesex Hospital | 1 | 3 | 6¾ |

"We see that at Middlesex Hospital more than *thirty* times as much alcoholic liquor was given as at University College—hospitals that are very near one another, and draw their patients from similar districts. Now there can be no reasonable excuse for that. If University College Hospital can treat its patients properly and successfully with so little alcohol there can be no doubt that Middlesex could do the same, and that a vast amount of money is thrown away there on drink. The money aspect of the question is, however, far the least. We grieve more over the effects of this large amount of drink on the patients and their friends, etc., etc. . . . . It may possibly be said that the larger expenditure at Middlesex is due to the cancer wards (this is only a surmise), where patients linger long under an exhausting disease. But this does not apply to St. Bartholomew's Hospital, where the expenditure is only 1s. 6¼d. less. The amount at the London Hospital is very small, only 1s. 7½d., and at Charing Cross not much more, 2s. 2¾d."

I confess to some little diffidence in venturing to criticise either the figures or the conclusions of Dr. J. J. Ridge in view of the vast intellectual superiority which we learn from further passages in the review before us, is possessed by the total abstainer over the mere "moderate drinker." Thus we learn "the power of judgment is abolished very early by alcohol." "The common use of alcohol lowers the average degree of self-control." And lastly, "The appeal must always be 'from Philip the moderate drinker to Philip the abstainer.'"

I think it better to say I am buoyed up by no hope of attaining to the lofty plane of reasoning on which the total abstainer weaves his statistics, but that I appeal merely to the vulgar herd of moderate drinkers whose ears are at least open to the language of facts, however limited their intelligence.

I should like in common with Dr. Ridge to point out "the great and extraordinary difference in the amount consumed at different hospitals," as given in Table A, but I find myself quite unable to endorse his "great and extraordinary" editorial remark that the expenditure "whether per bed or per patient does not appear, but is irrelevant for the present purpose."

Ordinary moderate drinkers will, I believe, be disposed to think it a very relevant matter in a comparison of expenditure to know whether the amount is spent upon one person alone or is divided up among 15 or 20. And a plain man might be excused for using plain language if he found he was treated to a set of statistics which placed in one table for purposes of comparison the cost of a varying number of patients so arranged as to convey the impression that the number provided was in each case identical.

The table of expenditure given by Dr. Ridge on Dr. Heywood Smith's authority is, as I have said, adapted only for the higher wits of the total abstainer. I subjoin a corrected version for the lower order of mind.

Table B.

| 1900. | Cost per patient. | Average yearly No. of patients to each bed. | Cost per bed. |
|---|---|---|---|
| University College Hospital | **8¾** | 17 | 12 4¾ |
| London Hospital | **1 7½** | 20 | 1 12 6 |
| Charing Cross Hospital | **2 2¾** | 15 | 1 13 5¼ |
| St. George's Hospital | **3 0¼** | 14 | 2 2 0 |
| King's College Hospital | 11½ | 14 | **13 6¾** |
| Guy's Hospital | 10 | 16 | **13 8¾** |
| Westminster Hospital | 11 | 15 | **13 11½** |
| St. Bartholomew's Hospital | 1 8 | 13 | **1 2 0** |
| The Middlesex Hospital | 1 9¾ | 13 | **1 3 6¾** |

Readers will kindly compare this table with that given by Dr. Ridge. They will then see that he has given for the first four hospitals the cost per patient; for the five next in order he has given the cost respectively of 14, 16, 15, and 13 patients. The number of patients provided for being wholly irrelevant, he is shocked to find that St. Bartholomew's and the Middlesex Hospital spend more on 13 patients than the London Hospital does on one.

I have used his own figures.

But these figures are not to our mind altogether to be relied on. They have been collected from the secretaries of various hospitals by an amateur investigator, who if he has any knowledge of what he is writing about has taken great and extraordinary pains to conceal it from his readers. He has laid his figures before the public in such a manner that I fear very few even of the total abstainers would have apprehended how the matter lay if I had not by the exercise of more than common ingenuity discovered how the trick was managed.

I prefer to quote a recognised authority in the matter readily accessible to all. The following figures from *Burdett's Hospital Annual* give the cost of alcohol per bed at the leading London hospitals in 1899. And it may be here observed, though that is perhaps a detail too small for the apprehension of Dr. Ridge's readers, that a comparison per bed is more satisfactory than a comparison per patient, as the average stay in hospital wards varies considerably from one hospital to another, and ordinary people are quite capable of understanding that a patient who remains in the hospital a month will cost a good deal more than one who is in residence only three weeks.

Table C.

| | Cost of Alcohol per Bed in 1899. £ | s. |
|---|---|---|
| London Hospital | 2 | 5 |
| Guy's Hospital | 0 | 10 |
| St. Bartholomew's Hospital | 1 | 15 |
| St. Thomas's Hospital | 1 | 5 |
| St. George's Hospital | 2 | 10 |
| The Middlesex Hospital | 1 | 10 |
| St. Mary's Hospital | 2 | 5 |
| King's College Hospital | 1 | 10 |
| University Hospital | 1 | 5 |
| Westminster Hospital | 2 | 0 |
| Charing Cross Hospital | 1 | 10 |
| The Royal Free Hospital | 1 | 0 |

Tables B and C refer to different years. I find, however, in referring to several previous years in Burdett's "Hospitals" that the average cost per bed for alcohol at the various hospitals mentioned is so equable that it would be far safer to abjure Dr. Heywood Smith's figures altogether. To give but one instance of error, the figures given for University College

Hospital by no means tally with the published report for the year 1900. The amount spent on alcohol that year was £157, and dividing this by 2,696, the total number of in-patients, it will be seen that the cost per patient works out at 1s. 2d., each instead of 8⅜d. It is little enough.

And now having delayed somewhat too long, perhaps over these grotesque statistics I may be permitted to go a step farther and attempt to grapple with Dr. Ridge's mournful conclusions with regard to them. "The money aspect of the question," he says, "is far the least." "We grieve more over the effects of this large amount of drink on the patients and their friends." An ordinary man, aware that his brain was to a certain extent disorganised by moderation in drink, might have sought guidance from the experts in such a matter before exposing his grief editorially to his readers. It might have occurred to him to consider to what extent he was justified in asserting that certain institutions were encouraging drunkenness within their walls and around them. Had he wanted information it is certain that he would have obtained it. Had he called at the Middlesex Hospital and said to the secretary, "How much alcohol do your patients get? I think you spend a good deal on it," that hard-worked individual would doubtless have told him all he wanted to know. But these methods are far too laborious for the quick-witted abstainer.

The *Review* informs us that the moderate use of alcohol decreases "the total output of energy." Nevertheless what its editor has failed to do the present writer has done, and the result may be briefly communicated. It has not been thought necessary to go through the sifting process in the case of each hospital, and as the Middlesex Hospital grieves Dr. Ridge most, it is to the Middlesex that I applied for information.

The total amount spent on alcohol at the Middlesex Hospital in 1900 was £475. Of this amount £139 was spent on beer for the household, including nurses. Reckoning that half the household are not beer-drinkers, this sum would suffice to provide the remainder with one glass of beer a day each.

We have still to account for an expenditure of £336, but of this £76 was spent on wine and spirits for the cancer wards. Of these patients 180 were received in 1900, and the average stay of each patient was 89 days. The amount spent on each patient was then almost exactly one penny a day. We have it on the authority of the *Medical Temperance Review* that the immediate effect of a stimulant is to induce "a feeling of *bien-être*." Is there anyone who would grudge such a sensation of *bien-être*, however transient, to sufferers from this distressing malady, in which perhaps the most pitiful of all the symptoms is the ceaseless sense of discomfort and general malaise? It is by no means clear to us that a penny a day can suffice to procure this small alleviation for the patients, and a little generous aid from subscribers might well be applied in this direction, seeing how wise a discretion is exercised with regard to stimulants by the medical staff of the hospital.

We have now left a sum of £260 expended on the remaining patients to the number of 3,578. But this must again be divided into £182 for wine and spirits, and £78 for malt liquor.

Let us take the malt liquor first. The beer costs 1s. a gallon; therefore each £1 will pay for 320 glasses. Again, we may surmise that at least half the patients never get any beer at all. The amount consumed is sufficient to give the remainder one glass of beer each for half the number of days they stay in hospital. Is this excessive?

The issue is now narrowed down to £182 spent on wine and spirits among 3,578 people, remaining on an average 26 days in hospitals. Divided among them all it works out at 1s. a head, or less than ½d. a day each. This would barely suffice to give a quarter of the number one spoonful of "drink" a day.

It may be thought that I am conferring a great deal more importance on the matter than the quality of the attack deserves.

It must be borne in mind, however, that this article was published shortly before Hospital Sunday, and circulated freely in a manner calculated to divert the benevolence of many persons away from the great medical charities which appeal for aid on that day. It has been obtruded, scored with blue pencil, and such epithets as "shameful" in the margin, under the notice of the institutions to which it refers, and I feel it an obligation to expose the slovenly and mischievous character of the figures sent out under the tacit sanction of 500 members of the medical profession.

The injury done to the hospitals by such an attempt at statistics is, however, in all probability not very considerable. "We grieve more," to quote the Editor's words once again, "over the effect" of this silly writing on the minds of medical men with regard to the great question of temperance. It is difficult to calculate the extent to which the advance of temperance is hindered by the intemperance of some of its advocates. Those of us who have made sacrifices of time and money and personal inclination in the effort to check what we all recognise to be a deep national disgrace are hampered at every turn by the exaggeration of the extremists.

In all decency let the members of the British Medical Temperance Association insist on a formal withdrawal and apology without a moment's delay.

